# Evidence for Sub-Haplogroup H5 of Mitochondrial DNA as a Risk Factor for Late Onset Alzheimer's Disease

**DOI:** 10.1371/journal.pone.0012037

**Published:** 2010-08-06

**Authors:** Aurelia Santoro, Valentina Balbi, Elisa Balducci, Chiara Pirazzini, Francesca Rosini, Francesca Tavano, Alessandro Achilli, Paola Siviero, Nadia Minicuci, Elena Bellavista, Michele Mishto, Stefano Salvioli, Francesca Marchegiani, Maurizio Cardelli, Fabiola Olivieri, Benedetta Nacmias, Andrea Maria Chiamenti, Luisa Benussi, Roberta Ghidoni, Giuseppina Rose, Carlo Gabelli, Giuliano Binetti, Sandro Sorbi, Gaetano Crepaldi, Giuseppe Passarino, Antonio Torroni, Claudio Franceschi

**Affiliations:** 1 Department of Experimental Pathology, University of Bologna, Bologna, Italy; 2 CIG-Interdepartmental Center for Biophysics and Biocomplexity Studies, University of Bologna, Bologna, Italy; 3 Department of Genetics and Microbiology, University of Pavia, Pavia, Italy; 4 Department of Cell and Environmental Biology, University of Perugia, Perugia, Italy; 5 National Council Research, Institute of Neuroscience, Padova, Italy; 6 Institute of Biochemistry, Medical Faculty Charité, Berlin, Germany; 7 Italian National Research Center for Aging (I.N.R.C.A.), Ancona, Italy; 8 Department of Molecular Pathology and Innovative Therapies, Polytechnic University of Marche, Ancona, Italy; 9 Department of Neurological and Psychiatric Sciences, University of Florence, Florence, Italy; 10 Regional Center for Cerebral Aging, Valdagno, Vicenza, Italy; 11 NeuroBioGen Lab-Memory Clinic, “Centro S.Giovanni di Dio-Fatebenefratelli”, Brescia, Italy; 12 Proteomics Unit, “Centro S.Giovanni di Dio-Fatebenefratelli”, Brescia, Italy; 13 Department of Cell Biology, University of Calabria, Rende, Cosenza, Italy; Mental Health Research Institute of Victoria, Australia

## Abstract

**Background:**

Alzheimer's Disease (AD) is the most common neurodegenerative disease and the leading cause of dementia among senile subjects. It has been proposed that AD can be caused by defects in mitochondrial oxidative phosphorylation. Given the fundamental contribution of the mitochondrial genome (mtDNA) for the respiratory chain, there have been a number of studies investigating the association between mtDNA inherited variants and multifactorial diseases, however no general consensus has been reached yet on the correlation between mtDNA haplogroups and AD.

**Methodology/Principal Findings:**

We applied for the first time a high resolution analysis (sequencing of displacement loop and restriction analysis of specific markers in the coding region of mtDNA) to investigate the possible association between mtDNA-inherited sequence variation and AD in 936 AD patients and 776 cognitively assessed normal controls from central and northern Italy. Among over 40 mtDNA sub-haplogroups analysed, we found that sub-haplogroup H5 is a risk factor for AD (OR = 1.85, 95% CI:1.04–3.23) in particular for females (OR = 2.19, 95% CI:1.06–4.51) and independently from the APOE genotype. Multivariate logistic regression revealed an interaction between H5 and age. When the whole sample is considered, the H5a subgroup of molecules, harboring the 4336 transition in the tRNA^Gln^ gene, already associated to AD in early studies, was about threefold more represented in AD patients than in controls (2.0% *vs* 0.8%; p = 0.031), and it might account for the increased frequency of H5 in AD patients (4.2% *vs* 2.3%). The complete re-sequencing of the 56 mtDNAs belonging to H5 revealed that AD patients showed a trend towards a higher number (p = 0.052) of sporadic mutations in tRNA and rRNA genes when compared with controls.

**Conclusions:**

Our results indicate that high resolution analysis of inherited mtDNA sequence variation can help in identifying both ancient polymorphisms defining sub-haplogroups and the accumulation of sporadic mutations associated with complex traits such as AD.

## Introduction

Alzheimer's Disease (AD) is the most common neurodegenerative disease and the leading cause of dementia among senile subjects. Few AD cases are early onset and familial, with autosomal dominant inheritance, while the majority of cases are late-onset (over 60 years old) and sporadic [1]. Sporadic AD (SAD) has a complex aetiology due to environmental and genetic factors which taken alone are not sufficient to cause the disease. Presently the major genetic risk factor in SAD is recognized in the allele e4 of apolipoprotein E (ApoE4). However, the causal factors in the majority of late-onset AD patients are still unknown and there is likely a complex interaction between genetic and environmental factors that eventually results in the pathology [2].

It has been proposed that SAD can be caused by defects in mitochondrial oxidative phosphorylation (OXPHOS) [3]. Structurally abnormal mitochondria have been observed in AD brains [4], and deficiencies in mitochondrial OXPHOS enzymes, such as cytocrome c oxidase, have been repeatedly reported in the brains and other tissues of AD patients [5], [6]. Mitochondria are deeply involved in various cellular processes such as ATP synthesis, heat production, reactive oxygen species (ROS) generation through oxidative phosphorylation, but also calcium signalling and apoptosis [7], [8]. Defects in OXPHOS inhibit ATP production, but also increase mitochondrial ROS production, which, in turn, can damage the mitochondrial genome (mtDNA). Thus, this hypothesis on AD pathogenesis claims that mitochondrial impairment may result from accumulated mtDNA damage that accompanies normal aging, amplified by disease-specific factors [9], [3]. Although there were reports of somatic mutations specifically at sites of known mtDNA regulatory elements, the available data from hybrid studies and post-mortem brain examinations did not provide conclusive proof that somatic mtDNA mutations can play a major or dominant role in AD aetiology [10], [11].

Human mtDNA codes for the 12S and 16S mitochondrial rRNAs, 22 tRNAs, and 13 polypeptides; these polypeptides are essential subunits of mitochondrial OXPHOS enzyme complexes, which generate the principal source of intracellular energy, ATP [12], [8]. It is inherited almost exclusively through the maternal lineage, does not recombine and is highly polymorphic. Mitochondrial genomes can be affiliated within haplogroups and sub-haplogroups on the basis of their specific sequence motifs, reflecting mutation events accumulated over time along diverging maternal lineages. These haplogroups tend to show a continental or regional localization, and a very large number of European, African, and Asian/Native American-specific haplogroups have been identified so far [13], [14].

Given the fundamental contribution of the mitochondrial genome for the respiratory chain, there have been a number of studies investigating the association between mtDNA lineages, aging and multifactorial diseases. It has been hypothesised that some mtDNA haplogroups are not neutral and have been selected through adaptation to climate and nutritional conditions [15], [8], [16]. Moreover, there are many reports on the association between mtDNA haplogroups and longevity [17]–[19], Leber hereditary optic neuropathy [20], as well as complex diseases like diabetes [21], ischaemic disease [22] and neurodegenerative diseases like Parkinson's Disease [23] and AD.

As for AD, contrasting data have been published. Some studies suggested that the transition at np 4336 of the mitochondrial tRNA glutamine gene is a risk factor for AD [24]–[26]. This mutation characterizes a European sub-branch of haplogroup H [13], which was termed H5a in more recent years [27], [28]. Later it has been reported that haplogroup T is under-represented whereas haplogroup J is over-represented in AD patients [29]. Haplogroup U has been reported to be under-represented in females and over-represented in male AD patients of European ancestry [30], while recently, it has been found that HV cluster is significantly associated with the risk of AD regardless of the gender and the APOE4 status [31]. On the other side, it is to note that several studies did not find any association between mtDNA haplogroups and AD by studying different European populations [32]–[34], [11], [35]. On the whole, no general consensus has been reached on the correlation between mtDNA haplogroups and AD [36].

Since AD is a common neurodegenerative disorder, affecting globally about 3% of people older than 60 years in the world [37], even a small risk associated with mtDNA haplotype could have major causative implications at a population level. It is therefore critically important to determine the role of mtDNA polymorphisms in AD by studying large cohorts of subjects at a level of molecular and phylogenetic resolution as fine as possible. Our study is then aimed to investigate the possible association of mtDNA at the sub-haplogroup level in a population of 936 AD patients and 776 controls from central-northern Italy, in order to clarify whether specific sub-haplogroup polymorphic sites are involved in AD, and to assess the possibility that associations detectable only at the sub-haplogroup resolution level might account for the discrepancies present in the literature regarding AD and European mtDNA inherited variability.

## Results

In our total sample of 1712 Italian subjects (936 AD patients and 776 controls) there is a higher proportion of female (72.9%) and APOE4+ (43.5%) subjects among AD patients relative to controls (60.6% and 13.1%, respectively). A separate comparison of genders further confirmed that APOE4 carriers were significantly different between AD patients and controls (**Table 1**).

**Table 1:** Characteristics of the study participants.

		AD patients (N = 936)	Controls (N = 776)	p-value
Age (years)	(mean±SD)	76.5±7.6	73.7±7.2	
Gender	Female	72.9%	60.6%	<0.0001
	Male	27.1%	39.4%	
APOE4 status	APOE4+	43.5%	13.1%	<0.0001
	APOE4-	56.5%	86.9%	
Female		(N = 682)	(N = 470)	
Age (years)	(mean±SD)	77.1±7.7	73.8±7.2	
APOE4 status	APOE4+	43.4%	11.1%	<0.0001
	APOE4-	56.6%	88.9%	
Male		(N = 254)	(N = 306)	
Age (years)	(mean±SD)	75.1±7.2	73.4±7.1	
APOE4 status	APOE4+	43.7%	16.3%	<0.0001
	APOE4-	56.3%	83.7%	

The hierarchical survey of diagnostic markers in the coding region allowed the classification of mtDNAs from patients and controls into more than 40 haplogroups/sub-haplogroups (**Table S1**). Most of these are typical of modern European populations, but a few East Asian (M) [38] and sub-Saharan African (L1b, L2a, L2c, L3d, L3e) [16], [39] mtDNAs were also detected. This latter finding is not unexpected, since low frequencies of African and East Asian haplogroups are not uncommon in populations of southern Europe. We then grouped, using a phylogenetic rationale whenever possible, all sub-haplogroups with frequencies lower than 1.5%, thus allowing a reduction of the overall number of categories from over 39 to 19.

No difference was found between AD patients and controls in the distribution of mtDNA groupings (**Table 2**). We further proceeded by comparing the 19 sub-haplogroup frequencies in AD patients and controls by taking into account the multiple comparison adjustments (p-value cut-off = 0.003); again no statistical difference was observed.

**Table 2:** Frequencies of mtDNA sub-haplogroups in AD patients and controls from Italy.

Sub-haplogroup^a^	AD patients			Controls		
	(N = 936)			(N = 776)		
	N	%	SE^b^	N	%	SE^b^
H*	182	19,4	0,0129	156	20,1	0,0144
H1	115	12,3	0,0107	97	12,5	0,0119
H3	28	3,0	0,0056	18	2,3	0,0054
H5^c^	39	4,2	0,0065	18	2,3	0,0054
H6	33	3,5	0,0060	21	2,7	0,0058
J1	64	6,8	0,0082	49	6,3	0,0087
J2	14	1,5	0,0040	13	1,7	0,0046
R0	46	4,9	0,0071	46	5,9	0,0085
T1	21	2,2	0,0048	17	2,2	0,0053
T2	84	9,0	0,0093	70	9,0	0,0103
U*	38	4,1	0,0065	32	4,1	0,0071
K	67	7,2	0,0084	68	8,8	0,0102
U4	23	2,5	0,0051	16	2,1	0,0051
U5a	46	4,9	0,0071	38	4,9	0,0077
U5b	18	1,9	0,0045	22	2,8	0,0060
V	31	3,3	0,0058	29	3,7	0,0068
W	20	2,1	0,0047	16	2,1	0,0051
X	26	2,8	0,0054	15	1,9	0,0049
Other	41	4,4	0,0067	35	4,5	0,0074

a Sub-haplogroups with frequencies lower than 1.5% were grouped. Thus R0 includes R0a, R1, HV*, HV0*, HV0a, HV1 and HV2; H* includes all mtDNAs belonging to haplogroup H, except those further classified (H1, H3, H5 and H6); the same rationale has been used for U*; whereas “Other” contains the heterogeneous haplogroups I, L1b, L2a, L2c, L3d, L3e, M, N and N1.

b Standard Error.

c Only for H5 there is a difference between AD patients and controls with a χ^2^ p-value (not adjusted for multiple comparisons) of 0.034.

Also when frequencies were compared separately for gender, we found no difference between AD patients and controls for the mtDNA, and overall no significant differences among the sub-haplogroup frequencies (**Tables S2 and S3**).

We then performed a univariate logistic regression analysis with the mtDNA variable to identify the potential sub-haplogroup associated with AD. Using as reference the haplogroup H, the only sub-haplogroup that resulted statistically associated with AD was H5 (OR = 1.89, 95%CI  = 1.03–3.42). Hence we created a dichotomous variable (H5 versus all other sub-haplogroups) and performed again the logistic regression analysis including sex, age and APOE4.Overall sub-haplogroup H5 has a slightly higher AD risk (OR = 2.19, 95%CI  = 1.06–4.51) in the female group than in the total sample (OR = 1.83, 95%CI  = 1.04–3.23). As expected, in our population APOE4 carriers have a higher AD risk than non-APOE4 carriers (OR = 5.08, 95%CI  = 3.98–6.50) and, among them, women have a higher risk than men (OR = 6.16, 95%CI  = 4.45–8.53, OR = 3.97, 95%CI  = 2.69–5.88, respectively).

Subsequently, mitochondrial sub-haplogroup H5 and the major acknowledged risk factors for AD (gender, age, APOE4) were compared in multivariate regression analysis. As shown in **Table 3**, gender (female), APOE4 allele, age and sub-haplogroup H5 are confirmed to be risk factor for AD. Moreover sub-haplogroup H5 interacts with age in modifying AD risk. Subjects younger than 75 years (corresponding to the median age in the total sample) and carrying sub-haplogroup H5 harbor a four-fold increased AD risk than subjects belonging to other sub-haplogroups, while among subjects who do not carry sub-haplogroup H5, those older than 75 years have a three-fold increased risk than younger subjects.

**Table 3:** Wald χ^2^ statistic (p-value), Odds ratio and 95% Confidence Intervals for factors associated with Alzheimer's disease in the entire sample.

		AD patients (N = 936) *vs* Controls (N = 776)^a^		
		p-value	OR	95%C.I.
Gender (Female)		<.0001	1.72	(1.37–2.16)
APOE4 status (APOE4+)		<.0001	5.54	(4.28–7.16)
AGE (years)^b^	Haplogroup			
≤75	Other		1.00	
	H5	0.0019	4.02	(1.67–9.66)
>75	Other		1.00	
	H5	0.5326	0.77	(0.33–1.77)
Haplogroup	AGE (years) b			
Other	≤75		1.00	
	>75	<.0001	3.12	(2.51–3.87)
H5	≤75		1.00	
	>75	0.3918	0.59	(0.18–1.96)

a Variables included in the model are age, gender, APOE4 and mtDNA haplogroup.

b Corresponds to median value.

We then performed the multivariate logistic regression by gender. In the female group APOE4 allele, age and sub-haplogroup H5 are confirmed to be risk factor for AD, and the APOE4 allele interacts with age in modulating AD risk. In female subjects aged 76 years (corresponding to the median age in the female sample) or younger, those carrying the APOE4 allele have a ten-fold increased AD risk than non APOE4 carriers, while within subjects older than 76 years, those carrying the APOE4 allele have a nearly four-fold increased AD risk than non-APOE4 carriers. Among female non-APOE4 carriers, those older than 76 years have a four-fold increased AD risk than younger subjects (**Table 4**). In the male group only APOE4 allele (OR = 3.87, 95%CI  = 2.60–5.77) and age >74 years (OR = 2.15, 95%CI  = 1.51–3.07) are confirmed to be risk factors for AD (data not shown).

**Table 4:** Wald χ^2^ statistic (p-value), Odds ratio and 95% Confidence Intervals for factors associated with Alzheimer's disease in females.

		AD patients (N = 682) *vs* Controls (N = 470)^a^		
		p-value	OR	95%C.I.
mtDNA haplogroup (H5)		0.0327	2.38	(1.07–5.27)
AGE (years)^b^	APOE4 status			
≤76	APOE4-		1.00	
	APOE4+	<.0001	9.56	(6.13–14.89)
>76	APOE4-		1.00	
	APOE4+	<.0001	3.70	(2.26–6.08)
APOE4 status	AGE (years)^b^			
APOE4-	≤76		1.00	
	>76	<.0001	3.90	(2.91–5.24)
APOE4+	≤76		1.00	
	>76	0.1744	1.51	(0.83–2.74)

a Variables included in the model are age, gender, APOE4 and mtDNA haplogroup.

b Corresponds to median value.

**Figure 1:**
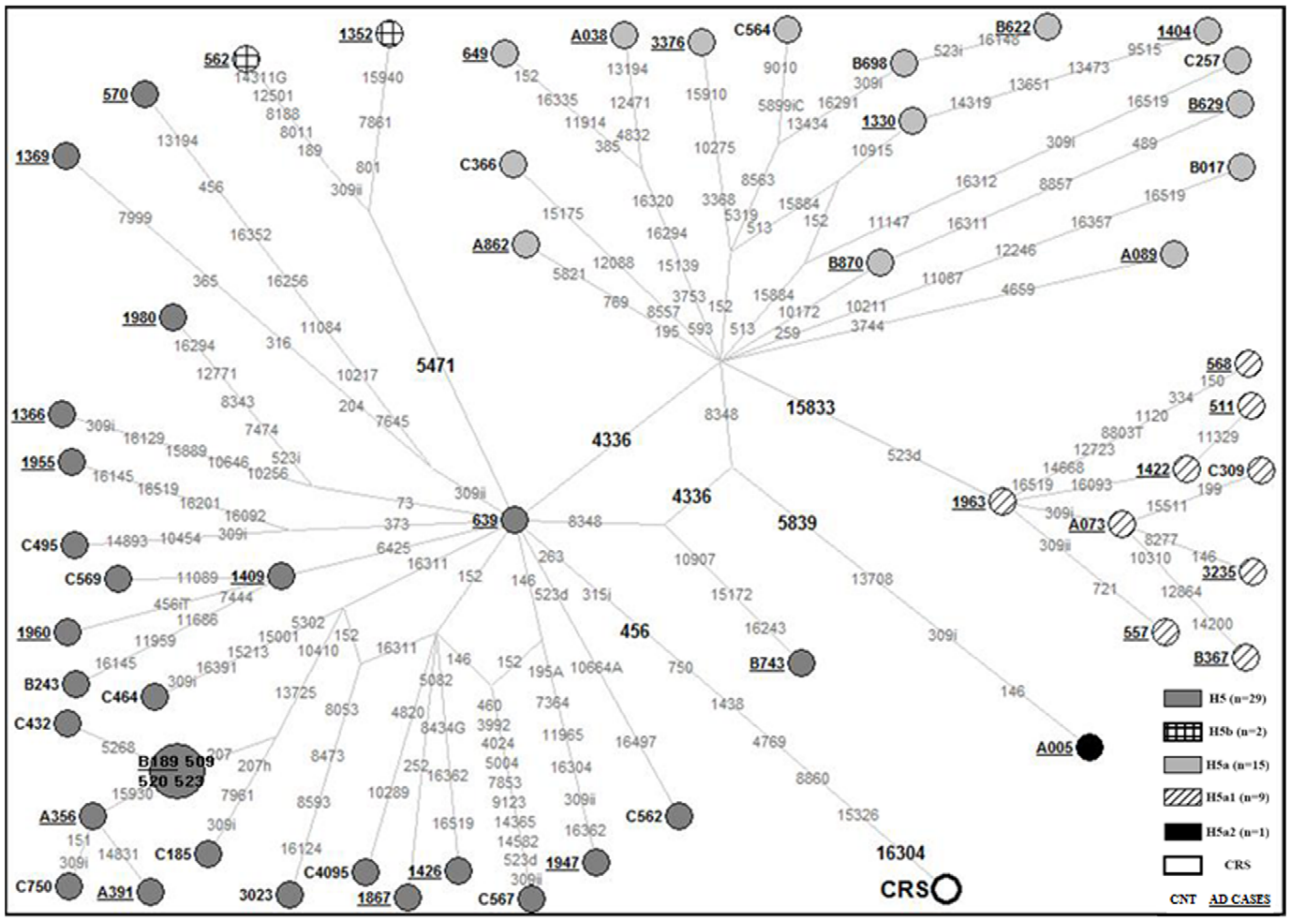
Phylogenetic network of the sub-haplogroup H5 complete sequences. Phylogenetic network based on the variation of 56 mtDNA complete sequences from Italian subjects (GenBank accession numbers: GQ983055–GQ983110) relative to the revised Cambridge Reference Sequence (rCRS, GenBank accession number: NC_012920). Numbers beside the nodes denote samples (Cases and Control subjects). Unless marked otherwise, the polymorphic variants, shown on the lines connecting the nodes, are transitions. Letters following the polymorphic variants indicate transversions or insertions (i) or deletions (d). Positions in bold are H5 specific mutations. We assigned a weight  = 10 to shared positions among all the samples and a weight  = 30 to the rare mutations in the D-loop region, while we assigned a weight  = 70 to shared positions and a weight  = 90 to the rare variants in the coding region.

To determine whether the increased risk associated with sub-haplogroup H5 could be attributed to specific mutations or mutational motifs, the complete sequence of virtually all H5 mtDNAs (56 out of 57; 38 AD patients and 18 controls) in our collection was determined and the phylogenetic distribution among the samples was analyzed. The results of the phylogenetic distribution and of the sequence analysis are summarized in **Table 5** **and** **Figure 1**. A total of 159 mutated positions relative to the reference sequence [40] were detected and analyzed in the network, including 104 nucleotide changes in the coding regions: the count of each kind of mutation is important in order to understand the mutational spectrum of the molecule and identify mutational hotspots. Overall 17 of the mutations (underlined and in italics in **Table 5**) were not previously reported in either MITOMAP (www.mitomap.org) or mtDB (www.genpat.uu.se/mtDB) and each mutation was observed only in a single H5 mtDNA.

**Table 5:** Mutations relative to the revised Cambridge reference sequences (rCRS) found in 56 mtDNAs belonging to sub-haplogroup H5.

Nucleotide Position^a^	Locus	Nucleotide Change	Aminoacid Change^b^	AD Patients: N = 38 (%)	Controls N = 18 (%)
73	D-loop (HVSII)	A>G	non coding	2 (5,3)	0 (0)
146	D-loop (HVSII)	T>C	non coding	3 (7,9)	1 (5,5)
150	D-loop (HVSII)	C>T	non coding	1 (2,6)	0 (0)
151	D-loop (HVSII)	C>T	non coding	0 (0)	1 (5,5)
152	D-loop (HVSII)	T>C	non coding	7 (18,4)	5 (27,78)
189	D-loop (HVSII)	A>G	non coding	1 (2,6)	0 (0)
195	D-loop (HVSII)	T>C	non coding	1 (2,6)	0 (0)
195A	D-loop (HVSII)	T>A	non coding	1 (2,6)	0 (0)
199	D-loop (HVSII)	T>C	non coding	0 (0)	1 (5,5)
204	D-loop (HVSII)	T>C	non coding	1 (2,6)	0 (0)
207	D-loop (HVSII)	G>A	non coding	5 (13,2)	4 (22,2)
252	D-loop (HVSII)	T>C	non coding	1 (2,6)	0 (0)
259	D-loop (HVSII)	A>G	non coding	0 (0)	1 (5,5)
263	D-loop (HVSII)	A>G	non coding	38 (100)	18 (100)
309+C	D-loop (HVSII)	ins	non coding	7 (18,4)	7 (38,9)
309+CC	D-loop (HVSII)	ins	non coding	5 (13,2)	1 (5,5)
315+C	D-loop (HVSII)	ins	non coding	38 (100)	18 (100)
316	D-loop (HVSII)	G>A	non coding	1 (2,6)	0 (0)
334	D-loop (HVSII)	T>C	non coding	1 (2,6)	0 (0)
*365*	D-loop (HVSII)	A>G	non coding	1 (2,6)	0 (0)
373	D-loop	A>G	non coding	1 (2,6)	1 (5,5)
385	D-loop	A>G	non coding	1 (2,6)	0 (0)
456	D-loop (HVSIII)	C>T	non coding	37 (97,4)	18 (100)
*456+T*	D-loop (HVSIII)	ins	non coding	1 (2,6)	0 (0)
460	D-loop (HVSIII)	T>C	non coding	0 (0)	1 (5,5)
489	D-loop (HVSIII)	T>C	non coding	1 (2,6)	0 (0)
513	D-loop (HVSIII)	G>A	non coding	2 (5,3)	1 (5,5)
523_524 delCA	D-loop (HVSIII)	del	non coding	8 (21,1)	3 (16,7)
523_524+CA	D-loop (HVSIII)	ins	non coding	2 (5,3)	0 (0)
593	tRNA phenylalanine	T>C	-	2 (5,3)	0 (0)
721	rRNA 12S	T>C	-	1 (2,6)	0 (0)
750	rRNA 12S	A>G	-	38 (100)	18 (100)
769	rRNA 12S	G>A	-	1 (2,6)	0 (0)
*801*	rRNA 12S	A>G	-	1 (2,6)	0 (0)
*1120*	rRNA 12S	C>T	-	1 (2,6)	0 (0)
1438	rRNA 12S	A>G	-	38 (100)	18 (100)
**3368**	NADH dehydrogenase subunit 1	T>C	Met NP > Thr P	1 (2,6)	0 (0)
3744	NADH dehydrogenase subunit 1	A>G	synonymous	1 (2,6)	0 (0)
3753	NADH dehydrogenase subunit 1	T>C	synonymous	2 (5,3)	0 (0)
**3992**	NADH dehydrogenase subunit 1	C>T	Thr P > Met NP	0 (0)	1 (5,5)
**4024**	NADH dehydrogenase subunit 1	A>G	Thr P > Ala NP	0 (0)	1 (5,5)
4336	tRNA glutamine	T>C	-	19 (50)	6 (33,3)
**4659**	NADH dehydrogenase subunit 2	G>A	Ala NP > Thr P	1 (2,6)	0 (0)
4769	NADH dehydrogenase subunit 2	A>G	synonymous	38 (100)	18 (100)
4820	NADH dehydrogenase subunit 2	G>A	synonymous	0 (0)	1 (5,5)
*4832*	NADH dehydrogenase subunit 2	C>T	synonymous	1 (2,6)	0 (0)
5004	NADH dehydrogenase subunit 2	T>C	synonymous	0 (0)	1 (5,5)
*5082*	NADH dehydrogenase subunit 2	T>C	synonymous	1 (2,6)	0 (0)
**5268**	NADH dehydrogenase subunit 2	A>G	Ile NP > Val NP	0 (0)	1 (5,5)
**5302**	NADH dehydrogenase subunit 2	T>C	Ile NP > Thr P	0 (0)	1 (5,5)
**5319**	NADH dehydrogenase subunit 2	A>G	Thr P > Ala NP	1 (2,6)	2 (11,1)
5471	NADH dehydrogenase subunit 2	G>A	synonymous	2 (5,3)	0 (0)
5821	tRNA cysteine	G>A	-	1 (2,6)	0 (0)
5839	tRNA tyrosine	C>T	-	1 (2,6)	0 (0)
5899+C	-	ins	-	0 (0)	1 (5,5)
6425	Cytochrome c oxidase subunit 1	T>C	synonymous	2 (5,3)	2 (11,1)
7364	Cytochrome c oxidase subunit 1	A>G	synonymous	1 (2,6)	0 (0)
**7444**	Cytochrome c oxidase subunit 1	G>A	STOP > Lys	0 (0)	1 (5,5)
*7474*	tRNA serine	A>G	-	1 (2,6)	0 (0)
7645	Cytochrome c oxidase subunit 2	C>T	synonymous	1 (2,6)	0 (0)
**7853**	Cytochrome c oxidase subinit 2	G>A	Val NP > Ile NP	0 (0)	1 (5,5)
7861	Cytochrome oxidase subunit 2	T>C	synonymous	1 (2,6)	0 (0)
7961	Cytochrome c oxidase subunit 2	T>C	synonymous	0 (0)	1 (5,5)
7999	Cytochrome oxidase subunit 2	T>C	synonymous	1 (2,6)	0 (0)
*8011*	Cytochrome c oxidase subunit 2	A>G	synonymous	1 (2,6)	0 (0)
8053	Cytochrome c oxidase subinit 2	A>G	synonymous	0 (0)	1 (5,5)
8188	Cytochrome c oxidase subunit 2	A>G	synonymous	1 (2,6)	0 (0)
8277	-	T>C	-	1 (2,6)	0 (0)
8343	tRNA lysine	A>G	-	1 (2,6)	0 (0)
8348	tRNA lysine	A>G	-	2 (5,3)	0 (0)
***8434G***	ATP synthase F0 subunit 8	C>G	Ile NP > Met NP	1 (2,6)	0 (0)
8473	ATP synthase F0 subunit 8	T>C	synonymous	0 (0)	1 (5,5)
**8557**	ATP synthase F0 subunit 6	G>A	Ala NP > Thr P	0 (0)	1 (5,5)
**8563**	ATP synthase F0 subunit 6	A>G	Thr P > Ala NP	1 (2,6)	2 (11,1)
***8593***	ATP synthase F0 subunit 6	A>G	Ile NP >Val NP	0 (0)	1 (5,5)
**8803T**	ATP synthase F0 subunit 6	A>T	Thr P > Ser P	1 (2,6)	0 (0)
**8857**	ATP synthase F0 subunit 6	G>A	Gly NP > Ser P	1 (2,6)	0 (0)
**8860**	ATP synthase F0 subunit 6	A>G	Thr P > Ala NP	38 (100)	18 (100)
**9010**	ATP synthase F0 subunit 6	G>A	Ala NP > Thr P	0 (0)	1 (5,5)
9123	ATP synthase F0 subunit 6	G>A	synonymous	0 (0)	1 (5,5)
*9515*	Cytochrome c oxidase subunit 3	C>T	synonymous	1 (2,6)	0 (0)
10172	NADH dehydrogenase subunit 3	G>A	synonymous	2 (5,3)	0 (0)
10211	NADH dehydrogenase subunit 3	C>T	synonymous	0 (0)	1 (5,5)
10217	NADH dehydrogenase subunit 3	A>G	synonymous	1 (2,6)	0 (0)
10256	NADH dehydrogenase subunit 3	T>C	synonymous	1 (2,6)	0 (0)
10275	NADH dehydrogenase subunit 3	T>C	synonymous	1 (2,6)	0 (0)
10289	NADH dehydrogenase subunit 3	A>G	synonymous	0 (0)	1 (5,5)
10310	NADH dehydrogenase subunit 3	G>A	synonymous	1 (2,6)	0 (0)
10410	tRNA arginine	T>C	-	5 (13,2)	4 (22,2)
10454	tRNA arginine	T>C	-	0 (0)	1 (5,5)
10646	NADH dehydrogenase subunit 4L	G>A	synonymous	1 (2,6)	0 (0)
*10664A*	NADH dehydrogenase subunit 4L	C>A	synonymous	0 (0)	1 (5,5)
**10907**	NADH dehydrogenase subunit 4	T>C	Phe NP > Leu NP	1 (2,6)	0 (0)
10915	NADH dehydrogenase subunit 4	T>C	synonymous	2 (5,3)	0 (0)
**11084**	NADH dehydrogenase subunit 4	A>G	Thr P > Ala NP	1 (2,6)	0 (0)
**11087**	NADH dehydrogenase subunit 4	T>C	Phe NP > Leu NP	0 (0)	1 (5,5)
11089	NADH dehydrogenase subunit 4	C>T	synonymous	0 (0)	1 (5,5)
11147	NADH dehydrogenase subunit 4	T>C	synonymous	0 (0)	1 (5,5)
11329	NADH dehydrogenase subunit 4	A>G	synonymous	1 (2,6)	0 (0)
*11686*	NADH dehydrogenase subunit 4	C>T	synonymous	0 (0)	1 (5,5)
11914	NADH dehydrogenase subunit 4	G>A	synonymous	1 (2,6)	0 (0)
11959	NADH dehydrogenase subunit 4	A>G	synonymous	0 (0)	1 (5,5)
*11965*	NADH dehydrogenase subunit 4	C>T	synonymous	1 (2,6)	0 (0)
12088	NADH dehydrogenase subunit 4	C>T	synonymous	0 (0)	1 (5,5)
12246	tRNA serine2	C>T	-	0 (0)	1 (5,5)
12471	NADH dehydrogenase subunit 5	T>C	synonymous	1 (2,6)	0 (0)
12501	NADH dehydrogenase subunit 5	G>A	synonymous	1 (2,6)	0 (0)
12723	NADH dehydrogenase subunit 5	A>G	synonymous	1 (2,6)	0 (0)
12771	NADH dehydrogenase subunit 5	G>A	synonymous	1 (2,6)	0 (0)
12864	NADH dehydrogenase subunit 5	T>C	synonymous	1 (2,6)	0 (0)
13194	NADH dehydrogenase subunit 5	G>A	synonymous	2 (5,3)	0 (0)
13434	NADH dehydrogenase subunit 5	A>G	synonymous	1 (2,6)	1 (5,5)
*13473*	NADH dehydrogenase subunit 5	A>G	synonymous	1 (2,6)	0 (0)
**13651**	NADH dehydrogenase subunit 5	A>G	Thr P > Ala NP	1 (2,6)	0 (0)
**13708**	NADH dehydrogenase subunit 5	G>A	Ala NP > Thr P	1 (2,6)	0 (0)
13725	NADH dehydrogenase subunit 5	C>T	synonymous	5 (13,2)	4 (22,2)
14200	NADH dehydrogenase subunit 6	T>C	synonymous	1 (2,6)	0 (0)
***14311G***	NADH dehydrogenase subunit 6	T>G	Tyr NP > Asp A	1 (2,6)	0 (0)
**14319**	NADH dehydrogenase subunit 6	T>C	Asn P > Asp A	1 (2,6)	0 (0)
14365	NADH dehydrogenase subunit 6	C>T	synonymous	0 (0)	1 (5,5)
**14582**	NADH dehydrogenase subunit 6	A>G	Val NP > Ala NP	0 (0)	1 (5,5)
14668	NADH dehydrogenase subunit 6	C>T	synonymous	1 (2,6)	0 (0)
**14831**	Cytochrome b	G>A	Ala NP > Thr P	1 (2,6)	0 (0)
14893	Cytochrome b	A>G	synonymous	0 (0)	1 (5,5)
15001	Cytochrome b	T>C	synonymous	0 (0)	1 (5,5)
15139	Cytochrome b	T>C	synonymous	2 (5,3)	0 (0)
15172	Cytochrome b	G>A	synonymous	1 (2,6)	0 (0)
*15175*	Cytochrome b	C>T	synonymous	0 (0)	1 (5,5)
**15213**	Cytochrome b	T>C	Ile NP > Thr P	0 (0)	1 (5,5)
**15326**	Cytochrome b	A>G	Tyr NP > Asp A	38 (100)	18 (100)
15511	Cytochrome b	T>C	synonymous	0 (0)	1 (5,5)
15833	Cytochrome b	C>T	synonymous	8 (21,1)	1 (5,5)
**15884**	Cytochrome b	G>A	Ala NP >Thr P	2 (5,3)	1 (5,5)
15889	tRNA threonine	T>C	-	1 (2,6)	0 (0)
15910	tRNA threonine	C>T	-	1 (2,6)	0 (0)
15930	D-loop	G>A	non coding	2 (5,3)	1 (5,5)
15940	D-loop	T>C	non coding	1 (2,6)	0 (0)
16092	D-loop (HVSI)	T>C	non coding	1 (2,6)	0 (0)
16093	D-loop (HVSI)	T>C	non coding	2 (5,3)	0 (0)
16124	D-loop (HVSI)	T>C	non coding	0 (0)	1 (5,5)
16129	D-loop (HVSI)	G>A	non coding	1 (2,6)	0 (0)
16145	D-loop (HVSI)	G>A	non coding	1 (2,6)	1 (5,5)
16148	D-loop (HVSI)	C>T	non coding	1 (2,6)	0 (0)
16201	D-loop (HVSI)	C>T	non coding	1 (2,6)	0 (0)
16243	D-loop (HVSI)	T>C	non coding	1 (2,6)	0 (0)
16256	D-loop (HVSI)	C>T	non coding	1 (2,6)	0 (0)
16291	D-loop (HVSI)	C>T	non coding	1 (2,6)	1 (5,5)
16294	D-loop (HVSI)	C>T	non coding	3 (7,9)	0 (0)
16304	D-loop (HVSI)	T>C	non coding	37 (97,4)	18 (100)
16311	D-loop (HVSI)	T>C	non coding	6 (15,8)	6 (33,3)
16312	D-loop (HVSI)	A>G	non coding	0 (0)	1 (5,5)
16320	D-loop (HVSI)	C>T	non coding	2 (5,3)	0 (0)
16335	D-loop (HVSI)	A>G	non coding	1 (2,6)	0 (0)
16352	D-loop (HVSI)	T>C	non coding	1 (2,6)	0 (0)
16357	D-loop (HVSI)	T>C	non coding	0 (0)	1 (5,5)
16362	D-loop (HVSI)	T>C	non coding	2 (5,3)	0 (0)
16391	D-loop	G>A	non coding	0 (0)	1 (5,5)
16497	D-loop	A>G	non coding	0 (0)	1 (5,5)
16519	D-loop	T>C	non coding	3 (7,9)	2 (11,1)

a Novel mutations are underlined and in italics.

b Mutations resulting in an aminoacid change are in bold. NP (Not Polar), P (Polar), A (Acid).

The topology of our mtDNA network matches published European trees in most respects [41].The network data set included 38 AD cases and 18 healthy controls, belonging to H5. A reduced-median network was constructed based on the sequence variations in the entire mtDNA molecules and by placing the rCRS as reference (**Figure 1**).

The H5 network turned out to be star-like and constituted by a number of sub-clusters. The major dichotomy is due to the presence/absence of the 4336 mutation. The presence of this mutation characterizes H5a (N = 15) the cluster over represented in AD patients (1.1% in patients vs. 0.6% in controls). Moreover, entire mitochondrial sequence data show that H5a can be further subdivided; in particular there is one sub-branch termed H5a1 and defined by the mutation 15833 which encompasses many of mtDNAs within H5a (9 out of 15).

Twenty-nine of the mutations (in bold) listed in **Table 5** result in amino acid changes, but excluding the mutations 8860 and 15326 (rCRS private mutations), none was particularly common. The highest frequencies were indeed reached by the mutations 5319 and 8563, each observed in one AD patient (2.6%) and two controls (11.1%).

Among all mutations observed in the 56 H5 mtDNAs, the mutated position at 15833, defining the H5a1 subgroup, was observed in the 21.1% of the H5 AD patients and in the 5.5% of controls, when compared with the entire sample this difference was statistical significant (0.9% *vs* 0.1%; p = 0.039). The nucleotide change at np 4336 in the tRNA glutamine gene, already associated to AD in previous studies [24], [25], was observed in 50.0% of the H5 AD patients and 33.3% of the controls. When the whole sample is considered, the H5a subgroup of molecules, harboring the 4336 transition, was about threefold more represented in AD patients than in controls (2.0% *vs* 0.8%; p = 0.031), and it might account for the H5 frequency increase in AD patients (4.2% *vs* 2.3%).

We did not find any statistical significant result when comparing the number of mutations along the mtDNA molecule by mtDNA regions (**Table 6**). Subsequently, we searched for groups of singleton mutations falling in specific mtDNA regions that may increase the susceptibility to AD. In particular we focused on singleton mutations falling in tRNA and rRNA genes, considering that previous studies have shown that mutations in these genes, including the transition at np 4336 (tRNA^Gln^) and the G3196A mutation (16S rRNA), may be associated with AD [24], [42]. We found that ten sporadic mutations (single occurrences) were present in the tRNA plus rRNA genes from AD mtDNA sequences, while only two sporadic mutations were found in the same genes from controls. We verified the significance of this finding by comparing the number of the tRNA+rRNA mutations with the number of mutations falling in the remaining of coding region (43 in AD patients and 37 in controls). The χ^2^ test, under the hypothesis of a homogeneous distribution of mutations showed a tenuous significance (p = 0.05).

**Table 6:** Number of mutations found along the mtDNA molecule regions in AD patients (N = 936) *vs* controls (N = 776).

mtDNA region	N. mutations in AD (N = 534)	N. mutations in CNT (N = 261)
**D-loop**	**235**	**115**
ND1	4	2
ND2	44	24
COI	3	3
COII	5	3
ATPase8	1	1
ATPase6	41	24
COIII	1	0
ND3	6	2
ND4L	1	1
ND4	7	6
ND5	16	5
ND6	4	2
CytB	52	25
**Total (genes)**	**185**	**98**
Y	1	0
R	5	5
T	2	0
C	1	0
Q	19	6
K	3	0
F	2	0
S	1	0
S2	0	1
**Total (tRNA)**	**34**	**12**
12S	80	36
**Total (rRNA)**	**80**	**36**

## Discussion

Although there is large amount of evidence about the role of mitochondria in the pathogenesis of AD, a definitive conclusion regarding the association of mtDNA haplogroup with AD has not been reached yet [36]. To date several studies reported lack of association between mtDNA haplogroups and AD [32]–[34], [11], [35], while others found positive associations with specific haplogroups as well as specific haplogroup clusters analyzing different Caucasian populations [24], [29]–[31], [43].

To our knowledge, this is the first case-control study investigating, at the sub-haplogroup level, the association of mtDNA with Alzheimer's Disease. We analyzed a large cohort of AD patients (N = 936) and controls (N = 776) comparable for age and ethnicity from the central-northern regions of Italy, where the controls were directly assessed for their cognitive status. We found that sub-haplogroup H5 appears to be associated with a higher risk of AD in both the total sample and the female group. This result was in keeping with a recent study on the Polish population which revealed that the super-haplogroup HV and haplogroup H are associated with a higher risk of AD [31].

Studies of both survival after sepsis [44] and sperm motility [45] have shown significant associations with mtDNA haplogroups, leading to the proposal that mitochondria bearing haplogroup H mtDNAs, and particularly haplogroups H3, H4, H5 and H6 [46] are associated with a more tightly coupled oxidative phosphorylation, and consequently they should lead to an increased production of ROS than those with haplogroup T. Accordingly, it was hypothesized that subjects with haplogroup H could be more prone to oxidative stress than those with other haplogroups [8], and consequently more susceptible to neurodegenerative diseases, in which oxidative stress plays a major role. This hypothesis would account for the higher AD risk we observed for H5 subjects.

We also found that sub-haplogroup H5 interacts with age in modifying AD risk. H5 subjects younger than 75 years old have a higher AD risk than non-H5 subjects, while in those older than 75 years no increased risk is observed. Age is a strong risk factor for AD [37] and this interaction suggests that age 75, corresponding to the median age in our sample, could be considered as a threshold, under which risk factors such as sub-haplogroup H5 and APOE, could independently exert their major effect on the development of the disease, while over this age value other risk factors, such as aging itself, would largely prevail.

The presence of mtDNA sub-haplogroup H5 confers a greater risk of AD to women than to the total sample of AD patients considered. In our sample the proportion of AD females was higher than in the female control group, however we can exclude that the association is merely due to a gender imbalance because the frequencies of all haplogroups in AD females were comparable to the female controls (and also to male controls), these latter being similar to the northern Italian general population [47].

In males we did not find any significant association but only a trend toward an increase in H5 frequency in AD males relative to controls. It is to note that males are about one third of our total AD sample, because they are less represented both among the elderly [48] and in AD patients [49]. Thus, the negative association between H5 sub-haplogroup and AD in men could be explained by the smaller size of the AD male group. However, a specific or more pronounced risk in women cannot at present be excluded, given the growing evidence that components of the AD phenotype differ significantly based on gender [50].

As for APOE, any association emerged between sub-haplogroup mtDNA variation and APOE4 genotype, suggesting that they likely exert an independent effect on AD. Previous results on this topic are discordant, some Authors reporting a positive association [43], [31], while others no association [30], [35] between haplogroups and APOE4. Indeed, we found a strong association of APOE4 with age, this effect being particularly evident in females younger than 76 years, i.e. the same age group where mtDNA sub-haplogroup H5 appears to exert its major influence on AD.

In order to assess if the H5 background of the patients harboured mutations responsible for the increased AD risk, we investigated the complete mtDNA sequences of our H5 subjects. We did not find any particular mutational pattern in the H5 patients (compared to controls) except for a threefold increase of the H5 subgroup (H5a) characterized by a transition at position 4336 (tRNA^Gln^ gene) that has been previously shown to be associated with AD [24], [25], and a sevenfold increase of the synonymous mutation at np 15833 which defines the H5a1 sub-haplogroup. The latter finding is difficult to explain unless that the high frequency of this C>T transition at np 15833 in AD patients is due to a mutational bias toward specific codon usage at synonymous sites [16].

In addition sporadic mutations (showing up in one sample only) are slightly more numerous in tRNA plus rRNA genes of AD samples than in controls. This finding is also in keeping with growing evidences showing that mutations in the protein synthesis machinery of mitochondria may be frequent cause of human degenerative diseases [42]. Thus our results confirm the relationship between mtDNA sequence variation and AD and suggest that both ancient polymorphisms defining sub-haplogroups and the accumulation of sporadic mutations are most likely involved.

Further study will need to better focus on the genetic variation falling in tRNA and rRNA genes, that is the mitochondrial translation machinery. In fact a better characterization of the molecular problems caused by these mutations in combination with may be of help in characterizing different subgroups of sporadic AD patients and help in their treatment.

Summarizing, the limitation of the present study is the lack of a replication study in another population. Accordingly, we cannot exclude population-specific effects. However, the large number of patients and controls in our study allows us to largely exclude possible false positive results. A replica of our study is quite demanding, owing to difficulties in enrolling another comparable large number of patients and of cognitively well assessed controls either in the same geographic area or in other European countries. The strengths are: (i) our study refers to a number of clinically well assessed AD patients (936) and controls (776) which is quite consistent; (ii) both AD patients and controls have been recruited in a specific relatively large geographic area thus avoiding possible bias related to a founder effect or population heterogeneity; (iii) the control subjects have been tested for their cognitive capability; (iv) we have identified for the first time a candidate risk sub-haplogroup for AD which extends previous data regarding H-V cluster on a more limited number of AD patients and controls in a Polish population [31]; (v) we have provided 56 new mtDNA complete sequences all referring to the H5 sub-haplogroup which not only suggest that sporadic mutations in mitochondrial tRNA and rRNA genes could be involved in AD risk but also and more in general represent a major contribution on mtDNA genetics regarding this specific haplogroup in a Caucasian population; (vi) we have pointed out the relationship between H5 mtDNA sub-haplogroup and other well known risk factors for AD, such as APOE4 genetic polymorphism and age. These data allowed us to identify the subgroup of AD patients (younger than 75 years of age; female sex) where H5 sub-haplogroup has a stronger effect.

## Methods

DNA samples from 1712 (936 AD patients and 776 healthy controls) unrelated central-northern Italian subjects were analyzed. The geographic origin and the number of samples for each participating group were the following: Lombardy (405 patients; 192 controls), Veneto (248 patients; 414 controls), Tuscany (138 patients; 45 controls), Marche (145 patients; 125 controls). All subjects were of Italian descent.

Patients were diagnosed by skilled evaluation units as suffering from probable AD, according to NINCDS-ADRDA criteria [51] and underwent a comprehensive geriatric assessment, including an extended neuropsychological evaluation.

The control group was comparable for age, sex and ethnicity to the patient group. A major characteristic of this control group is that they were directly assessed by MMSE in order to exclude subjects affected by cognitive deficiency. Written informed consent was obtained from all control individuals and primary caregivers on behalf of AD Patients. Written informed consent was obtained from all control individuals and primary caregivers on behalf of AD Patients. Each Institution which provided the DNA samples received the approval from their own ethical committees. In particular the Ethic committees of the: Italian National Research Center for Ageing in Ancona, Department of Neurological and Psychiatric Sciences of University of Florence, Regional Center for Cerebral Ageing in Valdagno and the S. Giovanni di Dio-Fatebenefratelli Center in Brescia have given their approval.

DNA was recovered from fresh blood by phenol-chloroform standard procedures. MtDNA profiles in patients and controls were determined by sequencing the entire mtDNA control region for each subject from nucleotide position (np) 16024 to np 576. This was followed by a hierarchical survey of haplogroup and sub-haplogroup diagnostic markers in the coding region [20], [47].

APOE genotyping was performed as previously described [52]. Patients and controls were stratified into two subgroups, according to their APOE4 status: those carrying at least one APOE4 allele (*APOE4+*) and non-*APOE4* carriers (*APOE4-*).

Complete mtDNA sequences were obtained after treatment of total DNA samples with REPLI-g Mitochondrial DNA kit (Qiagen, Valencia, CA) for specific whole genome amplification of mtDNA, followed by PCR amplification with MitoALL Resequencing kit (Applera, Foster City, CA). The PCR products (5–10 ng) were purified by EXOSAPit (U.S. Biochemical, Cleveland, Ohio) and used for direct sequencing with BigDye kit version 3.1 (Applera). Electropherograms were inspected with SeqScape version 2.5 software (Applera). All the samples were aligned and compared with the revised Cambridge Reference Sequence (rCRS) [40].

Reduced median network analyses, based on nucleotide variation on the complete sequences, were carried out by Network 4.5.1.0 (http://fluxus-engineering.com) [53] with an e default value of zero.

Because of mutation rate heterogeneity in mtDNA there is not a consensus on the weights to be assigned to the mutated nucleotide positions. As reported in other studies [27], [54] and after experimental tests we choose to give different weights to nucleotide positions in the D-loop and coding regions of our samples. After an initial run, we summed the statistics (counts of each mutation) obtained for all samples and used them to weight the characters from 10 to 90 with an inverted linear relation against the number of occurrences in the statistics.

### Statistical Analysis

Mitochondrial sub-haplogroups, genotype frequencies and gender were compared between AD patients and controls using the χ^2^ or Fisher-exact test, while differences in age were investigated through the non parametric Wilcoxon rank-sum test.

The mitochondrial sub-haplogroup variability envisages at least 40 subgroups, which have been further grouped into 19 subgroups considering their frequencies and phylogenetic relationship.

The comparisons between each of the 19 mtDNA sub-haplogroups in AD patients and controls have been computed by applying both the Bonferroni's adjustments and the Holm's step procedure. All analyses were performed separately for women and men, as well as for the total group, and multivariate logistic regression analysis using a stepwise procedure, adjusting for significant interactions (considering a threshold level of 0.15), was used to assess the relationship between independent variables (mtDNA, APOE4 status, age and gender) and the outcome. Tests for statistical significance were two-sided with  = 0.05; effect size for the association was measured as odds ratio (OR) with 95% confidence intervals (CI). SAS (9.1) statistical software from SAS Institute, Cary (NC), was used for all statistical analyses.

### Web Resources

Accession numbers and the URL for data presented herein are as follows: GenBank, http://www.ncbi.nlm.nih.gov/Genbank/ (complete H5 sequences [accession numbers GQ983055–GQ983110])

MITOMAP: A Human Mitochondrial Genome Database. http://www.mitomap.org, 2009

mtDB: Human Mitochondrial Genome Database. www.genpat.uu.se/mtDB

Network: Free Phylogenetic Network Software. http://www.fluxus-engineering.com

## Supporting Information

Table S1 Frequencies of mtDNA sub-haplogroups in 936 AD patients and 776 controls from central-northern Italy.
(0.07 MB DOC)

Table S2 Frequencies of mtDNA sub-haplogroups in 682 female AD patients and 470 female controls from central-northern Italy.
(0.04 MB DOC)

Table S3 Frequencies of mtDNA sub-haplogroups in 254 male AD patients and 306 male controls from central-northern Italy.
(0.04 MB DOC)
