# Supplementary material for: Evidence for Sub-Haplogroup H5 of Mitochondrial DNA as a Risk Factor for Late Onset Alzheimer's Disease
Source: PLoS One. 2010 Aug 6;5(8):e12037. doi: 10.1371/journal.pone.0012037 (PMC2917370; doi:10.1371/journal.pone.0012037)
Supplement: Table S1 — Frequencies of mtDNA sub-haplogroups in 936 AD patients and 776 controls from central-northern Italy. (0.07 MB DOC) [file pone.0012037.s001.doc]

**Table S1.** **Frequencies of mtDNA sub-haplogroups in 936 AD patients and 776 controls from central-northern Italy**

| mtDNA  sub-haplogroups^a^ | AD patients | | | Controls | | |
| --- | --- | --- | --- | --- | --- | --- |
|  | (N=936) | | | (N=776) | | |
|  | N | % | SE | N | % | SE |
|  |  |  |  |  |  |  |
| H* | 182 | 19,4 | 0,0129 | 158 | 20,4 | 0,0145 |
| H1 | 115 | 12,3 | 0,0107 | 96 | 12,4 | 0,0118 |
| H3 | 28 | 3,0 | 0,0056 | 18 | 2,3 | 0,0054 |
| H5 | 39 | 4,2 | 0,0065 | 18 | 2,3 | 0,0054 |
| H6 | 33 | 3,5 | 0,0060 | 21 | 2,7 | 0,0058 |
| HV* | 11 | 1,2 | 0,0035 | 18 | 2,3 | 0,0054 |
| HV0* | 10 | 1,1 | 0,0034 | 8 | 1,0 | 0,0036 |
| HV0a | 4 | 0,4 | 0,0021 | 2 | 0,3 | 0,0018 |
| HV1 | 7 | 0,7 | 0,0028 | 3 | 0,4 | 0,0022 |
| HV2 | 1 | 0,1 | 0,0011 | 0 | 0,0 | 0,0000 |
| I | 10 | 1,1 | 0,0034 | 14 | 1,8 | 0,0048 |
| J1 | 64 | 6,8 | 0,0082 | 49 | 6,3 | 0,0087 |
| J2 | 14 | 1,5 | 0,0040 | 13 | 1,7 | 0,0046 |
| L1b | 2 | 0,2 | 0,0015 | 0 | 0,0 | 0,0000 |
| L2a | 4 | 0,4 | 0,0021 | 0 | 0,0 | 0,0000 |
| L2c | 1 | 0,1 | 0,0011 | 0 | 0,0 | 0,0000 |
| L3d | 3 | 0,3 | 0,0018 | 0 | 0,0 | 0,0000 |
| L3e | 0 | 0,0 | 0,0000 | 3 | 0,4 | 0,0022 |
| M | 10 | 1,1 | 0,0034 | 5 | 0,6 | 0,0029 |
| N | 1 | 0,1 | 0,0011 | 1 | 0,1 | 0,0013 |
| N1 | 9 | 1,0 | 0,0032 | 8 | 1,0 | 0,0036 |
| R0a | 13 | 1,4 | 0,0038 | 15 | 1,9 | 0,0049 |
| R1 | 0 | 0,0 | 0,0000 | 1 | 0,1 | 0,0013 |
| T1 | 21 | 2,2 | 0,0048 | 17 | 2,2 | 0,0053 |
| T2 | 84 | 9,0 | 0,0093 | 68 | 8,8 | 0,0102 |
| K | 67 | 7,2 | 0,0084 | 68 | 8,8 | 0,0102 |
| U2 | 3 | 0,3 | 0,0018 | 1 | 0,1 | 0,0013 |
| U2e | 12 | 1,3 | 0,0037 | 15 | 1,9 | 0,0049 |
| U3 | 11 | 1,2 | 0,0035 | 6 | 0,8 | 0,0031 |
| U4 | 23 | 2,5 | 0,0051 | 16 | 2,1 | 0,0051 |
| U5a | 46 | 4,9 | 0,0071 | 39 | 5,0 | 0,0078 |
| U5b | 18 | 1,9 | 0,0045 | 23 | 3,0 | 0,0061 |
| U6 | 2 | 0,2 | 0,0015 | 1 | 0,1 | 0,0013 |
| U7 | 1 | 0,1 | 0,0011 | 6 | 0,8 | 0,0031 |
| U8b | 4 | 0,4 | 0,0021 | 2 | 0,3 | 0,0018 |
| V | 31 | 3,3 | 0,0058 | 29 | 3,7 | 0,0068 |
| W | 20 | 2,1 | 0,0047 | 16 | 2,1 | 0,0051 |
| X | 26 | 2,8 | 0,0054 | 15 | 1,9 | 0,0049 |
| Other | 1 | 0,1 | 0,0011 | 2 | 0,3 | 0,0018 |

^a^H* includes all mtDNAs belonging to haplogroup H, except those further classified (H1, H3, H5 and H6). The same rationale has been used for HV* and HV0*.
