# Supplementary material for: Evidence for Sub-Haplogroup H5 of Mitochondrial DNA as a Risk Factor for Late Onset Alzheimer's Disease
Source: PLoS One. 2010 Aug 6;5(8):e12037. doi: 10.1371/journal.pone.0012037 (PMC2917370; doi:10.1371/journal.pone.0012037)
Supplement: Table S2 — Frequencies of mtDNA sub-haplogroups in 682 female AD patients and 470 female controls from central-northern Italy. (0.04 MB DOC) [file pone.0012037.s002.doc]

**Table S**2. Frequencies of mtDNA sub-haplogroups in 682 female AD patients and 470 female controls from central-northern Italy

| mtDNA  sub-haplogroups^a^ | AD patients | | | Controls | | |
| --- | --- | --- | --- | --- | --- | --- |
|  | (N=682) | | | (N=470) | | |
|  | N | % | SE | N | % | SE |
| H* | 126 | 18,5 | 0,0149 | 95 | 20,2 | 0,0185 |
| H1 | 82 | 12,0 | 0,0125 | 57 | 12,1 | 0,0151 |
| H3 | 15 | 2,2 | 0,0056 | 11 | 2,3 | 0,0070 |
| H5^b^ | 31 | 4,5 | 0,0080 | 10 | 2,1 | 0,0067 |
| H6 | 25 | 3,7 | 0,0072 | 15 | 3,2 | 0,0081 |
| J1 | 45 | 6,6 | 0,0095 | 29 | 6,2 | 0,0111 |
| J2 | 10 | 1,5 | 0,0046 | 10 | 2,1 | 0,0067 |
| R0 | 32 | 4,7 | 0,0081 | 25 | 5,3 | 0,0104 |
| T1 | 17 | 2,5 | 0,0060 | 14 | 3,0 | 0,0078 |
| T2 | 65 | 9,5 | 0,0112 | 45 | 9,6 | 0,0136 |
| U* | 29 | 4,3 | 0,0077 | 22 | 4,7 | 0,0097 |
| K | 54 | 7,9 | 0,0103 | 41 | 8,7 | 0,0130 |
| U5a | 37 | 5,4 | 0,0087 | 18 | 3,8 | 0,0089 |
| U5b | 14 | 2,1 | 0,0054 | 12 | 2,6 | 0,0073 |
| V | 25 | 3,7 | 0,0072 | 18 | 3,8 | 0,0089 |
| W | 15 | 2,2 | 0,0056 | 10 | 2,1 | 0,0067 |
| X | 19 | 2,8 | 0,0063 | 10 | 2,1 | 0,0067 |
| Other | 22 | 3,2 | 0,0068 | 19 | 4,0 | 0,0091 |

^a^Sub-haplogroups with frequencies lower than 1.5% were grouped. H* includes all mtDNAs belonging to haplogroup H, except those further classified (H1, H3, H5 and H6). The same rationale has been used for U*.

^b^For H5 there is a significant difference between AD patients and controls, with a χ^2^ p-value (not adjusted for multiple comparisons) of 0.03.
