# Supplementary material for: Evidence for Sub-Haplogroup H5 of Mitochondrial DNA as a Risk Factor for Late Onset Alzheimer's Disease
Source: PLoS One. 2010 Aug 6;5(8):e12037. doi: 10.1371/journal.pone.0012037 (PMC2917370; doi:10.1371/journal.pone.0012037)
Supplement: Table S3 — Frequencies of mtDNA sub-haplogroups in 254 male AD patients and 306 male controls from central-northern Italy. (0.04 MB DOC) [file pone.0012037.s003.doc]

**Table S3.** Frequencies of mtDNA sub-haplogroups in 254 male AD patients and 306 male controls from central-northern Italy

| mtDNA  sub-haplogroups^a^ | AD patients | | | Controls | | |
| --- | --- | --- | --- | --- | --- | --- |
|  | (N=254) | | | (N=306) | | |
|  | N | % | SE | N | % | SE |
| H* | 56 | 22,0 | 0,0260 | 63 | 20,6 | 0,0231 |
| H1 | 33 | 13,0 | 0,0211 | 39 | 12,7 | 0,0191 |
| H3 | 13 | 5,1 | 0,0138 | 7 | 2,3 | 0,0085 |
| H5 | 8 | 3,1 | 0,0110 | 8 | 2,6 | 0,0091 |
| H6 | 8 | 3,1 | 0,0110 | 6 | 2,0 | 0,0079 |
| J1 | 19 | 7,5 | 0,0165 | 20 | 6,5 | 0,0141 |
| J2 | 4 | 1,6 | 0,0078 | 3 | 1,0 | 0,0056 |
| R0 | 14 | 5,5 | 0,0143 | 21 | 6,9 | 0,0145 |
| T1 | 4 | 1,6 | 0,0078 | 3 | 1,0 | 0,0056 |
| T2 | 19 | 7,5 | 0,0165 | 23 | 7,5 | 0,0151 |
| U* | 9 | 3,5 | 0,0116 | 10 | 3,3 | 0,0102 |
| K | 13 | 5,1 | 0,0138 | 27 | 8,8 | 0,0162 |
| U5a | 9 | 3,5 | 0,0116 | 21 | 6,9 | 0,0145 |
| U5b | 4 | 1,6 | 0,0078 | 11 | 3,6 | 0,0106 |
| V | 6 | 2,4 | 0,0095 | 11 | 3,6 | 0,0106 |
| W | 5 | 2,0 | 0,0087 | 6 | 2,0 | 0,0079 |
| X | 7 | 2,8 | 0,0103 | 5 | 1,6 | 0,0072 |
| Other | 19 | 7,5 | 0,0165 | 15 | 4,9 | 0,0123 |

^a^Sub-haplogroups with frequencies lower than 1.5% were grouped. H* includes all mtDNAs belonging to haplogroup H, except those further classified (H1, H3, H5 and H6). The same rationale has been used for U*.
